# Superconductivity in PrNiO_2_ Infinite‐Layer Nickelates

**DOI:** 10.1002/adma.202416187

**Published:** 2025-03-10

**Authors:** Hoshang Sahib, Aravind Raji, Francesco Rosa, Giacomo Merzoni, Giacomo Ghiringhelli, Marco Salluzzo, Alexandre Gloter, Nathalie Viart, Daniele Preziosi

**Affiliations:** ^1^ Universitè de Strasbourg, CNRS IPCMS UMR 7504 Strasbourg F‐67034 France; ^2^ Laboratoire de Physique des Solides, CNRS Universite Paris‐Saclay Orsay 91405 France; ^3^ Synchrotron SOLEIL L'Orme des Merisiers BP 48 St Aubin Gif sur Yvette 91192 France; ^4^ Dipartimento di Fisica Politecnico di Milano Piazza Leonardo da Vinci 32 Milano I‐20133 Italy; ^5^ European XFEL Holzkoppel 4 D‐22869 Schenefeld Germany; ^6^ CNR‐SPIN Complesso di Monte S. Angelo via Cinthia ‐ I‐80126 Napoli Italy

**Keywords:** infinite‐layer nickelates, RIXS, STEM, superconductivity

## Abstract

Several reports about infinite‐layer nickelate thin films suggest that the superconducting critical temperature versus chemical doping phase diagram has a dome‐like shape, similar to cuprates. Here, a highly reproducible superconducting state in undoped PrNiO_2_ thin films grown on SrTiO_3_ are demonstrated. Scanning transmission electron microscopy measurements show coherent infinite‐layer phase with no visible stacking‐fault defects, an overall high structural quality where possible unintentional chemical doping or interstitial oxygen, if present, sum well below the measurable threshold of the technique. X‐ray absorption measurements show very sharp features at the Ni L_3,2_‐edges with a large linear dichroism, indicating the preferential hole occupation of Ni^1+^‐3$d_{x^{2}-y^{2}}$ orbitals in a square planar geometry. Resonant inelastic X‐ray scattering measurements reveal sharp magnon excitations of 200 meV energy at the magnetic Brillouin zone boundary, highly resonant at the Ni^1 +^ absorption peak. The results indicate that, when properly stabilized, infinite‐layer nickelate thin films are superconducting without chemical doping.

## Graphical abstract

This study unveils a superconducting state in undoped parent compound PrNiO_2_ thin films, challenging previous views on nickelate superconductivity. Through scanning transmission electron microscopy and spectroscopic techniques, the research demonstrates the high structural quality and distinct electronic properties of the thin films grown by pulsed laser deposition. While requiring an independent verification, this result suggests that the self‐doping mechanism is sufficient for superconductivity and the reported phase diagram of infinite‐layer nickelates might need a revision.

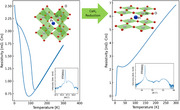

## 1 Introduction

Superconductivity in Infinite‐Layer (IL) nickelate thin films is under scrutiny since its discovery,^[^ ^1^ ^]^ and several crucial questions remain unanswered. Unlike cuprates, IL nickelates show no long‐range antiferromagnetic (AFM) order nor bulk superconductivity,^[^ ^2^ ^]^ suggesting that a template is strictly necessary to properly stabilize the IL phase. The latter is obtained by following a delicate synthesis process, which poses severe reproducibility problems.^[^ ^3^ ^]^ Because of the difficulties in optimizing the growth and reduction parameters, the electronic and magnetic properties of these new compounds have not yet been fully established. From the synthesis point of view, the presence of a capping layer emerged as a relevant step to reduce the defect density, although superconductivity was demonstrated in both capped^[^ ^1^ ^]^ and uncapped^[^ ^4^ ^]^ chemically doped IL films. In particular, the absence of a capping layer largely limits the quality of the samples.^[^ ^5^, ^6^ ^]^ Uncapped NdNiO_2_ IL nickelate are only partially reduced, and shows a (303) stripe‐organized interstitial oxygen pattern, which can be linked to the origin of the early observed charge ordering.^[^ ^7^ ^]^ Similarly, the bad‐metal low temperature resistance upturn observed in undoped IL nickelates, attributed to strong electron correlation effects,^[^ ^8^ ^]^ might not be an intrinsic property of the IL phase. In fact, NdNiO_2_ thin films realized by in situ oxygen deintercalation by atomic hydrogen did not show a pronounced low temperature resistance upturn.^[^ ^6^, ^9^ ^]^ So far, superconductivity in IL nickelates was reported by several groups only in chemically doped samples, regardless of the approaches used to stabilize the IL phase, including the solid state Al‐reduction method used in the case of Nd_1 − *x*_Eu_*x*_NiO_3_^[^ ^10^ ^]^ and recently for Pr_0.8_Ni_0.2_NiO_3_^[^ ^11^ ^]^ thin films or the atomic‐hydrogen method in Nd_1 − *x*_Sr_*x*_NiO_2_^[^ ^6^ ^]^ and La_1 − *x*_Sr_*x*_NiO_2_ thin films.^[^ ^12^ ^]^ In this context, it is worth mentioning that superconductivity was first demonstrated in La_0.8_Sr_0.2_NiO_2_ only after a significant optimization of the CaH_2_‐based process by Osada *et* *al*.^[^ ^13^ ^]^ In the same report, hints of a superconducting transition, at lower temperature, were provided also for undoped LaNiO_2_ thin films. So far, this result is not yet reproduced in other laboratories. Only very recently, an incomplete superconducting transition has been reported in the case of the undoped NdNiO_2_ films as well.^[^ ^14^ ^]^ The observations of superconductivity in undoped IL nickelates, while needing independent verification, suggest that the reported phase diagram of nickelates, also based on the notion that undoped compounds are weakly‐insulating bad‐metals,^[^ ^4^, ^15^, ^16^, ^17^ ^]^ might need a revision.

Here, we show that highly crystalline undoped PrNiO_2_ thin films are superconducting, with onset transition temperature (T_*C*_) in the 7–11 K range, and a zero resistance temperature up to 4 K. The highly reproducible zero resistance state is obtained via consecutive CaH_2_‐based topotactic reduction steps for samples prepared with a STO‐capping‐layer larger than 6 unit‐cells (uc), while all PrNiO_2_ films, irrespective of the presence/absence of a capping layer and/or the number of reduction cycles, show the onset of a superconducting transition. Scanning Transmission Electron Microscopy (STEM) shows very limited amount of defects and/or spurious phases in the entire observed volume, while divergence of the Center of Mass (dCOM) 4D‐STEM measurements reveals a complete absence of apical oxygens and NiO_2_ planes much more ordered than those of NdNiO_2_ samples.^[^ ^18^ ^]^ O K‐edge electron energy loss spectroscopy (EELS) further support the absence of any unintentional chemical doping. X‐ray Absorption Spectroscopy (XAS) at the Ni L_3,2_‐edges show very sharp peaks with a relatively large dichroism, demonstrating a complete reduction of our thin films.^[^ ^19^ ^]^ Additionally, Resonant Inelastic X‐ray Scattering (RIXS) shows that PrNiO_2_ are characterized by well defined magnons with a bandwidth of *ca*. 200 meV. Our results suggest that the superconductivity in undoped IL nickelates might be mostly hampered by subtle details accompanying the topotactic reduction process, instead of being controlled by a threshold value of chemical doping. The data show that PrNiO_2_ nickelates are self‐hole‐doped, as suggested by early theoretical reports.^[^ ^20^ ^]^ Finally, we show that the robust stabilization of the IL‐phase is a consequence of an high‐quality growth of the perovskite‐phase, which is an indispensable requisite for superconductivity in undoped infinite‐layer nickelates.[Supplementary-material adma202416187-supl-0001]

## 2 Results

**Figure 1:**
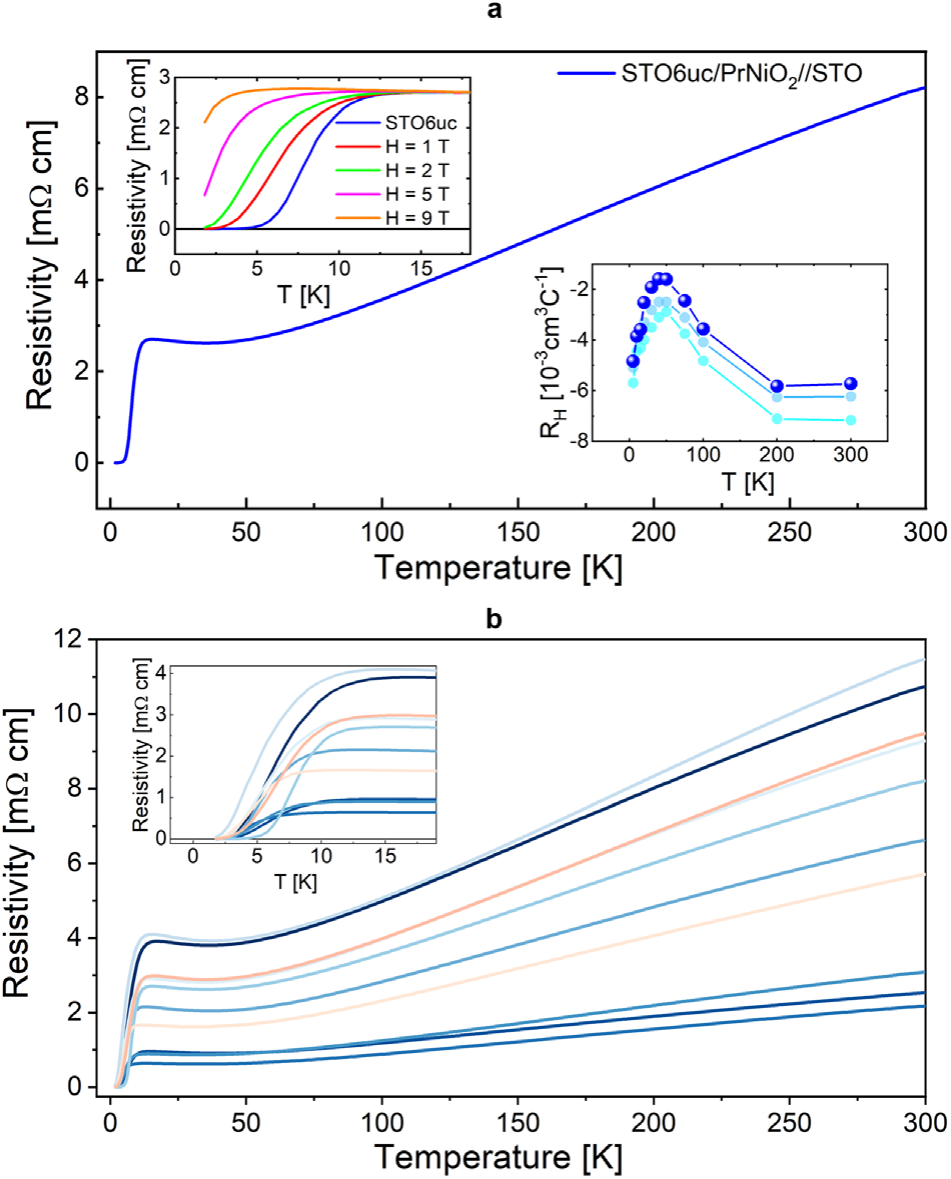
a) Temperature‐dependent resistivity of one of the STO(6uc)‐capped PNO2 film (16uc thick). Upper left inset shows the temperature dependence of the resistivity under applied magnetic fields; lower right inset displays the temperature‐dependent Hall coefficient for three nominally identical samples. b) Temperature‐dependent resistivity of several superconducting films grown in nominal similar conditions. The inset shows the same data around the superconducting transition. To note that samples with a lower residual resistance have a much broader superconducting transition.

Precursor perovskite PrNiO_3_ (PNO3) films were deposited onto SrTiO_3_ (STO) single crystal as substrate and capped by an epitaxial STO film grown in‐situ (See Experimental Section and Supporting Information). A correct cation stoichiometry is mandatory to reduce extended defects and to achieve bulk‐like transport properties, while granting a Ni^3+^ valence state.^[^ ^21^, ^22^ ^]^ It is well known that a large epitaxial mismatch can lower the energetic barrier for defects formation, that in the case of nickelates are usually identified as Ruddlesden–Popper (RP)‐like stacking faults.^[^ ^23^ ^]^ These type of defects largely hampered reproducible superconductivity in Nd‐based IL nickelate thin films, due to the lower crystallinity of the tensile strained perovskite precursor.^[^ ^24^ ^]^ According to the perovskite nickelates phase diagram, bulk PNO3 exhibits a less distorted unit cell akin to a larger ionic radius ($r_{Pr^{3+}}$ = 0.111 nm) compared to Nd ($r_{Nd^{3+}}$ = 0.110 nm).^[^ ^25^ ^]^ The increased Ni3*d*‐O2*p* orbital overlap leads to a metal‐to‐insulator transition (MIT) at a relatively lower temperature (*T*_*MIT*_ = 130 K), making the material more metallic compared to NdNiO_3_ (*T*_*MIT*_ = 200 K). The room temperature orthorhombic lattice parameters of bulk PNO3 are *a* = 0.541 nm, *b* = 0.538 nm, and *c* = 0.762 nm, corresponding to a pseudo‐cubic lattice with *a*_*pc*_ = 0.382 nm approximately.^[^ ^26^ ^]^ On the other hand, the PrNiO_2_ (PNO2) larger volume of the tetragonal (P4/mmm) unit cell (*a* = 0.394 nm and *c* = 0.328 nm)^[^ ^27^ ^]^ results in a higher compressive strain of PNO2 thin films onto STO (‐0.9%) compared to NdNiO_2_ (–0.5%). This should not be seen as a limiting factor for the stabilization of the IL phase, as there are reports of superconductivity in Nd_0.8_Sr_0.2_NiO_2_^[^ ^8^ ^]^ and Pr_0.8_Sr_0.2_NiO_2_^[^ ^28^ ^]^ thin films grown onto (LaAlO_3_)_0.3_(Sr_2_TaAlO_6_)_0.7_ (LSAT), with hints of an enhancement of the Tc by strain. Furthermore, calculations based on a dynamical vertex approximation technique predict that, under hydrostatic pressure, the PNO2 parent compound could become a high‐temperature superconductor by experiencing enhanced self‐doping of the Ni orbitals.^[^ ^29^ ^]^ We have used several experimental techniques to study the structural, electronic and magnetic properties of our fully reduced PNO2 samples. **Figure** **1**a displays the temperature dependence of the resistivity for one of our first superconducting PNO2 film, composed of 16 unit‐cells (uc)‐thick PNO2 film capped with 6 uc of STO (hereafter referred as STO6uc‐PNO2). Despite a relatively large resistivity at room temperature, compared to other reports in literature,^[^ ^16^, ^30^ ^]^ the metallic T‐linear behavior is followed by a slight upturn below *ca*. 50 K and a complete superconducting transition around 4 K. The measured onset critical temperature T_*C*_ (maximum curvature) is slightly below 11 K. This represents the main result of this work. In the top‐left inset of Figure 1a we show the temperature‐dependence of the resistivity as a function of the perpendicularly applied magnetic field, up to 9 Tesla. The normal state resistivity is insensitive to the magnetic field, while superconductivity is progressively suppressed. In the bottom‐right inset of Figure 1a, we show the temperature dependence of the Hall coefficient, which is negative in the whole temperature range measured for several nominally identical samples. The Hall coefficient shows a tendency toward a change of sign down to 50 K, and then an opposite trend. Nearby T_*C*_‐onset, its negative value further increases, which is remarkably different from change of sign, from negative to positive, observed in superconducting chemically doped samples.^[^ ^1^, ^16^, ^17^ ^]^

On the other hand, the Hall coefficient temperature dependence is very similar to what is measured in literature for undoped (non‐superconducting) and slightly doped NdNiO_2_, LaNiO_2_, and PrNiO_2_ infinite‐layer films.^[^ ^15^, ^16^, ^17^ ^]^

In Figure 1b we show the transport properties of a series of STO6uc‐PNO2 samples prepared in nominal similar conditions. All the samples display a zero resistance state below 4 K. The T_*C*_‐onset varies from sample‐to‐sample in a relatively small range, with a minimum value of 7 K and maximum value of 11 K, while the normal state residual resistivity and its value at room temperature varies in a much larger range, likely reflecting subtle details in the topotactic reduction process. The superconductivity in these undoped PNO2 films is very robust. In particular, an incomplete superconducting transition, with similar T_*C*_‐onset, is observed also in uncapped PNO2 samples, although a zero resistance state is obtained only on PNO2 capped with at least 6uc of STO (See Supporting Information for additional data). This confirms the key role played by the capping layer in stabilizing a clean and robust IL phase. We believe that the capping layer, beyond its role of protection against the direct contact with the CaH_2_ powder, allows to better maintain the compressive strain imposed by the substrate and necessary for the proper formation of the IL‐phase. A more systematic study investigating the precise effect of the capping layer on the stabilization of the infinite layer would be necessary to prove this hypothesis but it is beyond the current scope of this work.

**Figure 2:**
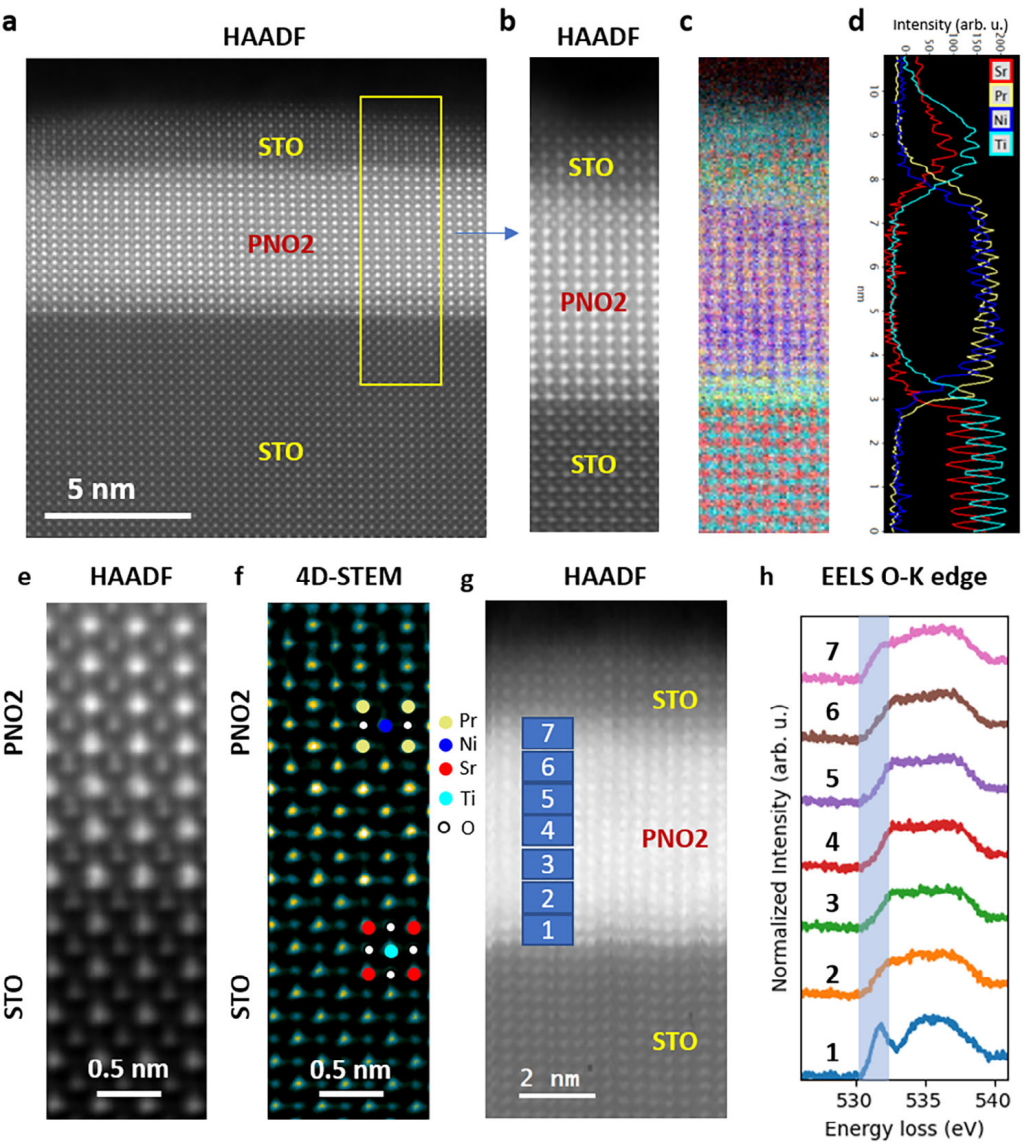
a) HAADF‐STEM image of a STO(6uc)–capped PNO2 film (16uc thick). b) HAADF image from a zoom‐in region. c) EELS Sr, Pr, Ni, and Ti elemental maps, and d) elemental profiles integrated over the zoom‐in region showing a Pr/Ni ratio close to 1 over the whole thin film. Both top and bottom interfaces are not sharp. e) A magnified HAADF image from the bottom interface region, f) the corresponding 4D‐STEM dCOM analysis showing clean infinite‐layer phase without the presence of any apical oxygen. h) Real space evolution of the O‐K edge EELS fine structure from regions as labeled in the HAADF image in (g).

We resorted to STEM measurements with a High‐Angle Annular Dark‐Field (HAADF) imaging technique and Electron‐Energy‐Loss Spectroscopy (EELS) to study the precise atomic stack, including both bottom and top interfaces. The atomic‐resolved HAADF‐STEM cross‐sectional image shown in **Figure** **2**a displays a clear infinite‐layer phase with a very high structural quality, thus demonstrating the overall absence of RP‐like defects in the precursor phase, as confirmed by HAADF‐STEM cross‐sectional images for STO6uc‐PNO3 samples. The EELS data acquired over the entire STO/PNO2//STO stack of a selected area of the HAADF image (Figure 2b), show a clear intermixing at the bottom film‐substrate and at the top film‐STO interfaces (Figure 2c). In particular, the interface with the TiO_2_‐terminated substrate is characterized by a one unit cell (Ni,Ti) intermixing layer, confirming other reports.^[^ ^31^, ^32^ ^]^ Additionally, the total element map intensity profile shown in Figure 2d clearly indicates that the top interface exhibits a complex Ni‐Pr‐Ti‐Sr atomic stack, which differs from other recent reports of more abrupt interfaces in Sr‐doped PNO3 and related IL thin films.^[^ ^32^ ^]^ It is worth to recall here that, early theoretical calculations proposed the formation of a quasi‐2D electron gas, with possible superconducting properties, in the case of a clean interface between the infinite‐layer nickelate and the STO substrate.^[^ ^33^ ^]^ Our HAADF‐STEM results, in agreement with Goodge *et* *al*., fully rule out the formation of a quasi‐2D electron gas since the intermixed (Ni,Ti)O_3_ interface‐layer fully compensates the polar discontinuity.^[^ ^31^ ^]^

Unintentional chemical doping can arise from Pr/Ni off stoichiometry, Sr‐diffusion and interstitial/apical oxygen incorporated into the nickelate layer. Our elemental EELS mapping shows a near unitary Pr/Ni ratio and no unintentional Sr‐doping (within the experimental resolution of the order of 10%) in the PNO2 sample. In order to identify any traces of these interstitial/apical oxygen, we employed 4D‐STEM dCOM imaging. Some of the authors have already shown that this powerful technique allows high‐resolution real space atomic mapping with good oxygen contrast.^[^ ^5^ ^]^ The HAADF image in Figure 2e, is obtained at the bottom interface, and the corresponding 4D‐STEM dCOM analysis in Figure 2f unveils the occupied oxygen sites not visible in the HAADF image. Excluding the intermixed interfacial unit‐cell, characterized by a (Ni,Ti)Ox composition in an octahedral coordination, we can easily distinguish occupied oxygen‐sites only within the NiO_2_ planes as, indeed, expected for a properly stabilized IL‐phase. This directly excludes traces of apical oxygen within the PNO2 samples and, moreover, demonstrates a rather clean Ni‐sites in square planar arrangement. To further evaluate the absence of any chemical source of doping with higher sensitivity, we performed atomically resolved EELS fine structure analysis at the Oxygen K‐edge. This is shown in Figure 2g,h. Figure 2h shows spatially resolved STEM‐EELS of O K‐edge fine structures in different regions of the sample, including the two interfacial regions. As expected for fully reduced IL,^[^ ^5^, ^34^ ^]^ the O K‐edge profiles do not show any pre‐peak feature, with the only exception of the area in proximity of the substrate, where a certain degree of Ni3*d*‐O2*p* hybridization is still observed because of the (Ni,Ti) intermixing layer,^[^ ^35^ ^]^ and nearby the top, intermixed, interface due to the STO‐capping‐layer. The absence of any pre‐edge is a clear indication that no unintentional chemical doping is present in our samples. In particular, a pre‐peak shoulder is always observed, also locally, in Sr‐doped IL‐nickelates, as shown in previous works.^[^ ^32^, ^34^, ^36^ ^]^ From this analysis, we can confidently conclude that the residual chemical doping is below the sensitivity of the multiple techniques used, and thus much lower than the minimum threshold needed so‐far to trigger superconductivity in chemically doped IL‐nickelates.

**Figure 3:**
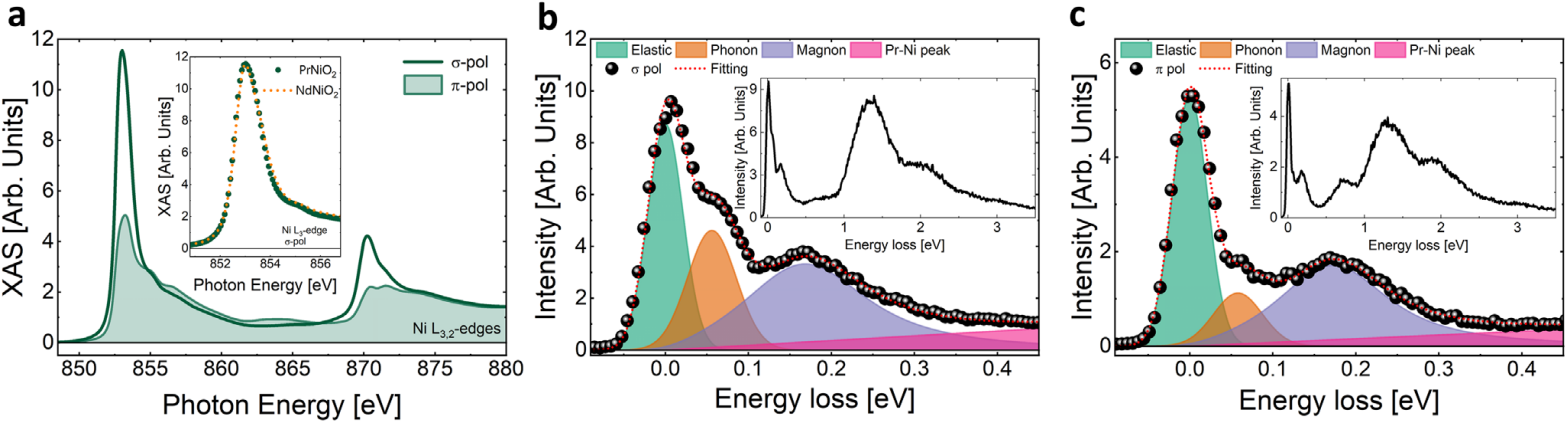
a) Linearly polarized XAS spectra at the Ni L_3,2_ edges for a STO6uc‐PNO2 sample, with electric field parallel (σ‐pol) and perpendicular (π‐pol) to the NiO_2_ planes. The inset shows the comparison with a XAS acquired in σ‐pol at the Ni L_3_‐edge for a NdNiO_2_ sample. b) Mid‐infrared region of the σ‐pol RIXS spectrum along the transferred momentum **Q** (‐0.36,0). The spectrum was obtained as a sum of three single spectra, with incident energies 852.3, 852.5, and 853.7 eV, to reduce the noise. The full‐range spectrum is reported in the inset. c) Mid‐infrared region of the π‐pol RIXS spectrum acquired at 852.5 eV photon energy. The spectrum was obtained as a sum of four single spectra, with exchange momentum H = 0.4, 0.425, 0.45, 0.475 r.l.u. along the [H,0] direction of the Brillouin zone, to reduce the noise. The shaded colorful areas in panels b‐c are the results of a fitting procedure as presented in Methods. All the measurements were performed at ca. T = 20 K.

In order to get information about the electronic structure of our superconducting PNO2 thin films, we performed XAS and RIXS measurements at the Ni L_3,2_‐edges and L_3_‐edge, respectively (see Experimental Section for details). Overall, the Ni L_3_ XAS spectra show features similar to properly optimized NdNiO_2_ thin films^[^ ^18^, ^19^, ^36^ ^]^ with a dominant peak due to the 2p^6^3d^9^ → 2p^5^3d^10^ transition. **Figure** **3**a shows XAS spectra acquired with linearly‐polarized light in the parallel and nearly perpendicular direction with respect to the NiO_2_ planes, *i*.*e*. σ‐pol and π‐pol, respectively. The more than 50% dichroism is a direct consequence of the very robust infinite‐layer phase, where the majority of the holes resides in the Ni^1+^‐3$d_{x^{2}-y^{2}}$ orbitals. These results confirm a full oxygen reduction after the topotactic process. Moreover, the Ni L_3_ peak is very sharp with a small shoulder at higher photon energy, speaking against adventitious sources of doping due to incomplete reduction (Ni^2+^) and/or excess oxygen.^[^ ^18^, ^36^ ^]^ For comparison, the inset in Figure 3a reports the XAS at the Ni L_3_‐edge peak acquired for a STO‐capped NdNiO_2_ with, indeed, very similar features. In Figure 3b,c we show the mid‐infrared region of RIXS spectra acquired in σ‐pol and π‐pol, respectively. We can easily recognize both the phonon and magnon excitations, which we fit with a Gaussian and a Damped Harmonic Oscillator susceptibility, respectively (please refer to the Supporting Information for further details about the fitting procedure). A linear background from the tails of higher‐energy excitation is also included. The phonon is apparently enhanced by σ incident polarization and, therefore, mostly associated to in‐plane Ni–O stretching modes, similarly to cuprates.^[^ ^37^ ^]^ The magnetic peak occupies an energy range around *ca*. 200 meV, consistently with previous results on IL nickelates.^[^ ^38^, ^39^, ^40^, ^41^ ^]^ In the insets we show the same RIXS spectra in the full energy loss range, enough to capture the Ni 3d orbital excitations between 1 and 3 eV.^[^ ^36^ ^]^ The peak at 0.6 eV, which is more pronounced in π‐pol, is attributed to out‐of‐plane Pr‐Ni hybridization, in analogy with other undoped nickelates.^[^ ^36^, ^39^, ^42^, ^43^ ^]^ Finally, the shape of the magnetic excitations, among the sharpest ever measured by RIXS in undoped IL nickelates, though not resolution‐limited, indicates that superconductivity is reached in the presence of long‐range spin‐spin correlations. This means that either a self doping mechanism is present, which does not perturb the antiferromagnetic background, or that superconductivity is obtained at extremely low levels of hole doping.

## 3 Discussion

The main finding of this study is the observation of a superconducting ground state in undoped PrNiO_2_ thin films: below, we briefly discuss our findings and their implications. The synthesis of infinite‐layer nickelates remains a real challenge, and one has to make sure that the reported superconductivity in nickelates parent compounds PNO2 thin films is not due to fortuitous sources of doping. There are at least three sources of possible unintentional chemical doping: adventitious Sr‐doping; Pr/Ni off‐stoichiometry; and excess oxygen due to incomplete de‐intercalation during the CaH_2_‐based topotactic reduction. The combination of STEM‐EELS, layer resolved O K‐edge EELS, and 4D‐STEM analysis on PNO2 samples in Figure 2, largely discussed in the previous section, clearly demonstrate that, locally, no relevant chemical‐doping could be detected. Moreover, XAS spectra (*cf*. to Figure 3a) show very sharp Ni^1+^ peaks, at odds with possible doping by chemical substitution, which will give signatures of Ni^2+^ features.^[^ ^18^ ^]^

**Figure 4:**
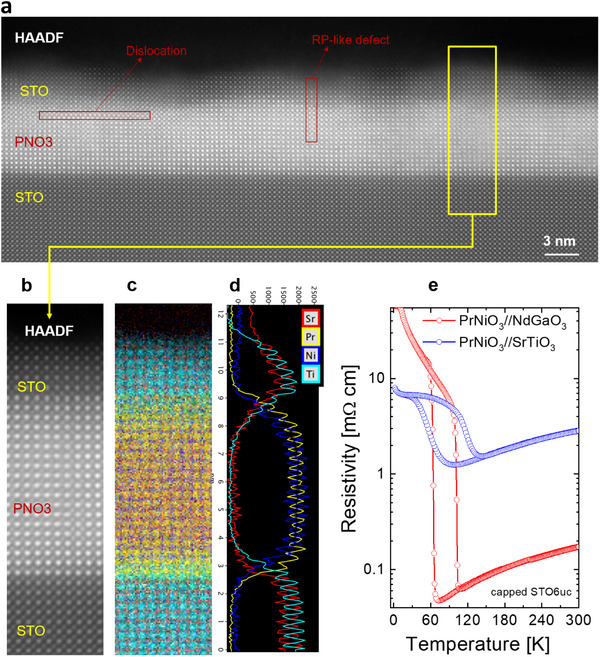
a) HAADF‐STEM image of the STO6uc‐PNO3 film. b) A defect‐free zoom‐in region. c) Elemental STEM‐EELS maps of Sr, Pr, Ni, and Ti. d) Elemental profiles integrated over the zoom‐in region showing a Pr/Ni ratio close to one throughout the investigated sample volume. Importantly, no Sr‐interdiffusion could be resolved. e) Temperature‐dependent resistivity of 16 unit‐cells thick PNO3 film grown onto STO and NdGaO_3_ substrates, and both capped with 6 unit‐cells of STO.

Here we discuss the relevance of the structural and chemical perfection of the perovskite precursor phase, which allows us to provide further support of the absence of a relevant unintentional chemical doping related to cation off stoichiometry. From the STEM‐EELS map on PNO2 in Figure 2, we found that the Pr/Ni ratio is close to one. However, to further exclude Pr/Ni off‐stoichiometry, in **Figure** **4**a–d we report atomic‐resolved HAADF‐STEM (Figure 4a,b) and EELS maps (Figure 4c,d) of our STO6uc‐PNO3 precursor samples. We find that the Pr/Ni ratio is equal to one within the experimental uncertainty, thus further excluding off‐stoichiometry issues. Additionally, in Figure 4e we report the transport properties of STO6uc‐PNO3 precursor samples deposited onto STO, and on well‐matched NdGaO_3_ (110) single crystal grown in the same deposition conditions, thus characterized by the same Pr/Ni composition.^[^ ^44^ ^]^ In these nickelates, the sharpness of the metal‐to‐insulator transition (MIT) is a valid proxy to gain major information about the Pr/Ni ratio.^[^ ^21^ ^]^ The data show a MIT with a reduced jump in the case of the PNO3//STO samples due to strain but still a very high MIT temperature, in agreement with other studies,^[^ ^16^ ^]^ while a very large resistance jump for the PNO3//NGO film, comparable to PrNiO_3_ single crystals.^[^ ^45^ ^]^ It is worth noting that the observation of a MIT in our precursor phase is a further indication of the absence of any Pr/Ni off‐stoichiometry and even Sr‐doping. Indeed, it is well known that the MIT is suppressed by a small amount of Pr/Ni off‐stoichiometry as well as by few percent of Sr‐doping. All these results imply a nearly unitary Pr/Ni ratio in our films, ruling out non‐unitary Pr/Ni ratio as a source of unintentional doping, and also Sr‐doping.

Beyond material imperfections, superconductivity in undoped nickelates, on the other hand, might be related to self‐doping of holes due to the R5*d*‐Ni3*d* hybridization. According to theoretical calculations, the R5*d* states gives rise to partially occupied electron pockets at the Γ‐point, and the hybridization with Ni‐3d*z* ^2^ states at the Fermi level provides self‐doped holes in the the quasi‐2D Ni‐3d*x* ^2^−*y* ^2^ derived band.^[^ ^20^ ^]^ This specific electronic configuration, while setting one of the major differences with the cuprates' fermiology, may be responsible of a superconducting state already at zero chemical doping.^[^ ^46^ ^]^

To summarize, we report clear experimental evidences that undoped PrNiO_2_ IL‐nickelates are superconducting, at odds with the observation of superconductivity only in IL doped by Sr and/or Ca cations, which provide extra‐holes in the NiO_2_ planes.^[^ ^1^, ^3^, ^16^, ^17^ ^]^ Our results, together with the report of the onset of superconductivity in undoped LaNiO_2_ thin films,^[^ ^13^ ^]^ and more recently in undoped NdNiO_2_ thin films,^[^ ^14^ ^]^ represent an important breakthrough for a better understanding of the infinite‐layer nickelates physics. According to these studies, due to the multi‐orbital nature of the electronic properties of nickelates, self‐doped holes in the Ni‐3d*x* ^2^−*y* ^2^ orbital‐derived bands, are enough to set a superconducting state. This requires a reconsideration of the IL nickelate phase‐diagram.

## 4 Experimental Section

### Thin Film Growth

The epitaxial growth of perovskite PrNiO_3_ films on 5 × 5 mm^2^ SrTiO_3_ substrates was performed by pulsed laser deposition using a 248 nm KrF excimer laser, with ceramic targets from Toshima Manufacturing Co. Ltd. The SrTiO_3_ substrates (Shinkosha Co. Ltd) were prepared by etching in an NH_4_F‐buffered HF solution, followed by annealing for 2 h at 950 °C in air to achieve a well‐defined TiO_2_‐terminated step‐terraced surface. Prior to growth, the substrate was pre‐annealed for 1 h at 800 °C under a pressure of 0.3 mbar in oxygen flow to ensure a very clean and sharp step‐and‐terrace surface. The layer‐by‐layer growth of the PrNiO_3_ was monitored via Reflection High Energy Electron Diffraction (RHEED) technique. The 15–20 unit cells thick PrNiO_3_ films were grown at a substrate temperature of 675 °C with an oxygen partial pressure $P_{O_{2}}$ of 0.3 mbar, using a laser fluence of 4 J/cm^2^ and a 1 × 1.4 mm^2^ laser spot size on the target.For capped samples, the SrTiO_3_ top layers (from 1 to 12 unit cell thickness), were grown at a substrate temperature of 575 °C and $P_{O_{2}}$ = 0.3 mbar, with a laser fluence of 1.3 J/cm^2^ and a 1 × 1.4 mm^2^ laser spot size. After growth, the samples were cooled to room temperature at a rate of 5 °C/min in the same oxidizing growth conditions.

### Topochemical Reduction (Infinite‐Layer Formation)

After growth, each sample was cut into two pieces of size 5 × 2.5 mm^2^ using a precision diamond wire saw (Well 3242). This method is used to cut the samples to avoid stress during the cutting process, which can introduce defects. The pieces of each sample to be reduced were placed in an evacuated silica tube sealed with a membrane valve, with 0.5 g of CaH_2_ powder in direct contact, as used in prior studies.^[^ ^47^ ^]^ The tube was heated to 260 °C at a rate of 5 °C/min, and held at this temperature for varying durations (2–8 hours) depending on the PrNiO_3_ and SrTiO_3_ capping thicknesses; then it was cooled to room temperature at a rate of 5 °C/min. The process was optimized through a systematic series of steps, with θ‐2θ scans and electrical transport measurements performed ex situ after each step to assess the degree of reduction. In particular, the highly reproducible zero resistance state is obtained via consecutive CaH_2_‐based topotactic reduction steps for samples prepared with a STO capping layer (from 3 up to 12 unit‐cells), while a superconducting transition is always encountered irrespective of the presence/absence of the capping layer and/or number of reduction cycles.

### Characterization

The surface morphology of the samples was examined using a Park XE7 (Park System) atomic force microscope (AFM) in true non‐contact mode. XRD measurements were performed using a Rigaku Smartlab diffractometer with a Cu‐Kα radiation source (0.154056 nm). For transport measurements, the 2.5 × 5 mm^2^ samples were wire‐bonded directly with Al, without the use of top‐electrodes. The wire bonds were placed at the four edges of the samples, and the measurements were conducted using the Van der Pauw method, with a current amplitude of 10 µA, with a cryo‐free Dynacool system (Quantum Design).

### High Resolution STEM‐EELS

The cross‐sectional focused ion beam (FIB) transmission electron microscopy (TEM) lamellae were prepared at C2N, University of Paris‐Saclay, France. Before FIB lamellae preparation, around 20 nm of amorphous carbon was deposited on top for protection. For additional protection, electron beam‐induced deposition of platinum and ion‐beam beam‐induced deposition of platinum were done. The HAADF imaging and EELS were carried out in a NION UltraSTEM 200 C3/C5‐corrected STEM. The experiments were done at 200 keV with a probe current of approximately 14 pA and convergence semi‐angles of 30 mrad. The EELS spectra were obtained using the full 4 × 1 configuration of a MerlinEM detector (Quantum Detectors Ltd) installed on a Gatan ENFINA spectrometer mounted on the microscope.^[^ ^48^ ^]^ The EELS spectrometer was set into non‐energy dispersive trajectories for 4D‐STEM experiments. Data was collected with only one chip of the MerlinEM detector in 6‐bit mode that enables faster acquisition without compromising on the signal‐to‐noise ratio. The high resolution EELS fine structure analysis was done in a monochromated and C3/C5 corrected NION Chromatem microscope operating at 100 keV, with a probe current of 30 pA, convergence semi‐angles of 25 mrad.

### XAS, RIXS

Measurements were performed at the ID32 soft x‐ray beamline of the ESRF, Grenoble, France. XAS spectra were measured at 10° grazing incidence, with linear polarization forming an angle of 0° or 80° with respect to the NiO_2_ planes, and labelled for brevity ∥NiO_2_ and ⊥NiO_2_, respectively. For RIXS, the combined resolution at the Ni L_3_‐edge was of 40 meV. The scattering angle 2θ was fixed at 149.5°. RIXS energy‐resolved maps were acquired at an incident angle of θ = 30° (grazing‐in geometry), corresponding to an exchanged in‐plane momentum of about 0.36 relative lattice units (r.l.u.). Momentum‐resolved maps were taken at the XAS resonance energy of 852.4 eV ca. in π incident polarization, which is known to enhance magnetic excitations. Grazing‐out geometry was adopted in this case, with incident angles θ between 80° and 140°. All RIXS and XAS measurements were performed at 20 K, the lowest temperature safely reachable by the ID32 cooling apparatus.

## Conflict of Interest

The authors declare no conflict of interest.

## Supporting information

Supporting Information

## Data Availability

The data that support the findings of this study are available from the corresponding author upon reasonable request.
