# Supplementary material for: Superconductivity in PrNiO2 Infinite‐Layer Nickelates
Source: Adv Mater. 2025 Mar 10;37(16):2416187. doi: 10.1002/adma.202416187 (PMC12016738; doi:10.1002/adma.202416187)
Supplement: Supplementary file 1 — Supporting Information [file ADMA-37-2416187-s001.pdf]

ADVANCED MATERIALS

Supporting Information

for *Adv. Mater.*, DOI 10.1002/adma.202416187

Superconductivity in $PrNiO_2$ Infinite-Layer Nickelates

Hoshang Sahib, Aravind Raji, Francesco Rosa, Giacomo Merzoni, Giacomo Ghiringhelli, Marco Salluzzo, Alexandre Gloter, Nathalie Viart and Daniele Preziosi**

Supporting Information

Superconductivity in $PrNiO_2$ infinite-layer nickelates

Hoshang Sahib[1], **Aravind Raji**[2,3], **Francesco Rosa**[4], **Giacomo Merzoni**[3,4], **Giacomo Ghiringhelli**[4], **Marco Salluzzo**[6], **Alexandre Gloter**[2], **Nathalie Viart**[1], **and Daniele Preziosi**[1]

[1] Université de Strasbourg, CNRS, IPCMS UMR 7504, F-67034 Strasbourg, France.
[2] Laboratoire de Physique des Solides, CNRS, Université Paris-Saclay, 91405 Orsay, France.
[3] Synchrotron SOLEIL, L'Orme des Merisiers, BP 48 St Aubin, 91192 Gif sur Yvette, France.
[4] Dipartimento di Fisica, Politecnico di Milano, Piazza Leonardo da Vinci 32, I-20133 Milano, Italy.
[5] European XFEL, Holzkoppel 4, Schenefeld, D-22869, Germany.
[6] CNR-SPIN Complesso di Monte S. Angelo, via Cinthia - I-80126 Napoli, Italy.

Growth of $SrTiO_3(d)/PrNiO_3//SrTiO_3$ heterostructures

In Figure S1a, we show the XRD θ-2θ symmetric scans of 16 unit cells (uc) thick PNO3//STO films capped with varying thicknesses (d) of STO, ranging from 0 to 12 uc. For all samples, intense (00ℓ) diffraction peaks ($\ell = 1, 2, 3$) are observed, confirming the high quality of the perovskite nickelate phase [2]. From the (00ℓ) peak positions, we calculate a c-axis lattice parameter of 0.375 nm, consistent with a fully strained and stoichiometric PNO3 thin film. Accounting for a Poisson ratio of 0.3, the expected out-of-plane lattice parameter for a tensile-strained PNO3 thin film onto STO is approximately of 0.375 nm, thus confidently ruling out any possible presence of off-stoichiometry and/or oxygen vacancies [3, 4]. A

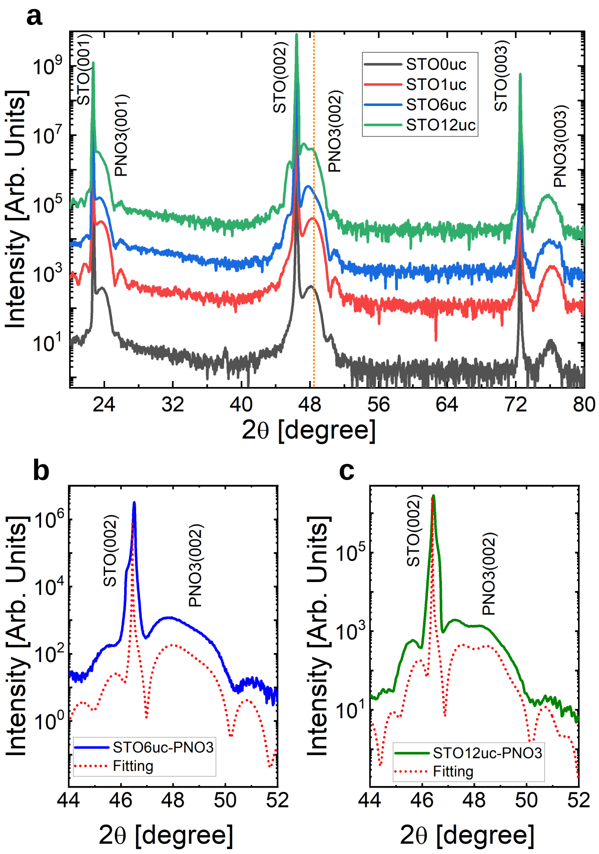

Figure S1 a) X-ray diffraction θ-2θ symmetric scans of PNO3 sample series with varying thicknesses of the STO capping layer. Below we show a zoomed-in around the (002) diffraction peak showing the peculiar modulation as described in the text together the fitting curves (red dotted lines), obtained by considering the presence of an epitaxial and coherent STO layer of 6uc (b) and (c) 12 uc as thickness. The fitting curves are obtained by using the software described in Ref. [1].

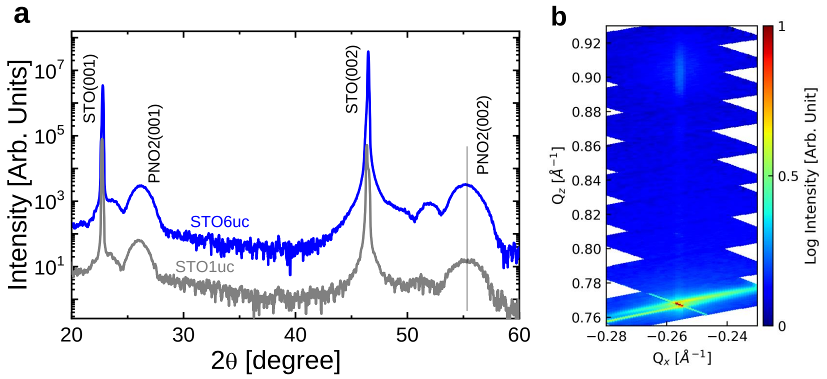

Figure S2 a) X-ray diffraction θ-2θ symmetric scans of STO6uc- and STO1uc-PNO2 samples, clearly showing the formation of the infinite-layer (IL) phase. b) Reciprocal space mapping performed around the asymmetric (103) STO diffraction peak, indicating that the samples are fully strained and exhibit a 0.333 nm c-axis parameter.

zoom-in around the (002) diffraction peaks (Figures S1b,c) reveals an intensity modulation of the peak profile for the PNO3 sample prepared with an STO capping layer thicker than 6uc. The HAADF-STEM results for the STO6uc sample (Figure 4b) clearly demonstrate that this intensity modulation of the (002) XRD peak profile is not due to secondary phases or hole-vacancies, but is most likely a direct consequence of the STO capping layer itself as, indeed, we carefully demonstrated by performing a fitting procedure by using a tool already used in literature [1]. The red dotted lines superimposed to the XRD pattern of our STO6uc-PNO3 and STO12uc-PNO3 samples properly reproduce the observed PNO3(002) XRD peak modulation.

Structural and transport properties of $SrTiO_3$(d)/PrNiO2//$SrTiO_3$ heterostructures

To stabilize the $PrNiO_2$ (PNO2) infinite-layer (IL) phase, we employed a CaH_2-based topotactic reduction process. Figure S2a shows the XRD θ-2θ symmetric scans of fully reduced samples capped with STO of 6 unit cells (STO6uc) and 1 unit cell (STO1uc), exhibiting robust (00ℓ) family peaks at the expected positions, with a c-axis of 0.333 nm, confirming the complete formation of the IL phase. Notably, features on the left of the main (00ℓ) diffraction peaks in the STO6uc-capped sample are likely attributed to Laue fringes, indicating the high quality of the IL phase, stabilized coherently by the appropriate thickness of the capping layer [5, 6]. Reciprocal Space Mapping (RSM) measurements around the asymmetric STO(-103) and PNO2(-103) diffraction peaks confirm that the PNO2 films are fully strained to the substrate and show no other features, as shown in Figure S2b. This is further corroborated by a geometrical phase analysis (GPA) map obtained via HAADF-STEM measurements, shown in Figure S3, which displays a homogeneous 15% reduction in the c-axis (GPA-ϵ_{xx}), supporting the macroscopic observations via XRD and indicating a fully strained IL-phase to the substrate (GPA-ϵ_{yy}).

In Figure S4 we show the transport properties of the full PNO2 series prepared with different STO(d) capping layer thicknesses (left), and also a series of PNO2(d) samples with the same STO6uc capping layer and overall similar conditions for the topotactic reduction. While the superconducting transition is always present, only samples with a capping layer thicker than 6 unit cells reach the zero resistance state. On the other side, the zero-resistance state in encountered only for sample 16uc thick. This largely demonstrates that the structural quality of the precursor phase (eventually worsened for a thicker sample) is a key parameter to obtain a superconducting state with a clear zero resistance state. This demonstrates, beyond the HAADF-STEM data, that the PNO2 superconductivity is not linked to any possible source of Sr interdiffusion.

Energy dependence of the magnon

As already suggested by the colour maps in Figure 3(b), the energy dependence of the single magnon follows a peculiar trend with respect to the other main spectral features (e.g., elastic peak, phonon). In particular, as shown in Fig. S5, the magnon intensity resonates before the absorption edge, $\simeq$ 0.4 eV de-tuned from the XAS peak, as opposed to the elastic and the phonon intensity. Moreover, the magnon resonance appears to be significantly narrower than the other two features presented in Fig. S5(b).

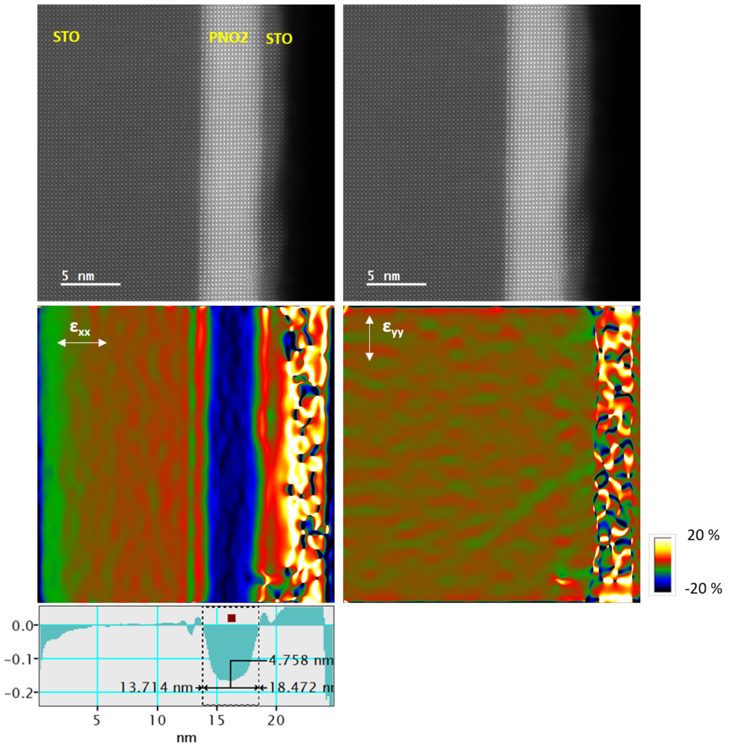

Figure S3 (Top) HAADF-STEM images for a PNO2 sample capped with 6 unit cells of STO, with (Bottom) GPA analysis applied, confirming a 15-16% reduction in the out-of-plane c-axis and full in-plane strain of the IL-phase. This reduction confirm the overall XRD result and shown in S2

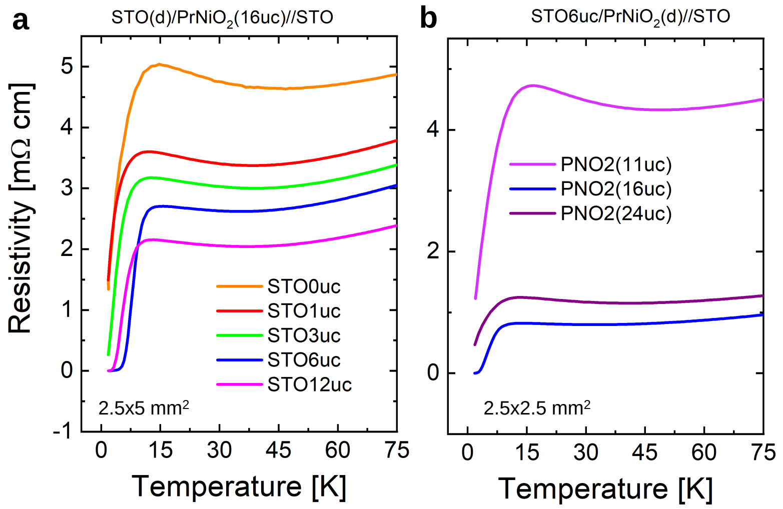

Figure S4 Temperature-dependent resistivity of the superconducting PNO2 sample series, prepared with (a) varying unit cells of the STO-capping layer but constant PNO2 thickness and (b) varying unit cells of PNO2 but constant STO-capping layer. The zero resistance state is achieved for samples with moderate PNO2 thickness (16 uc) and STO layers thicker than 6 unit cells.

Fitting of RIXS spectra

Here we introduce the fitting procedure for the results for the RIXS spectra showed in Figure 3c of the main text. A Damped Harmonic Oscillator susceptibility is employed for the magnetic peak, while two resolution-wide Gaussians fit the elastic and phonon contribution. The tail of the broad 0.6 eV peak due to Pr5d hybridization is taken into account with a linear background. Fittings close to the Gamma point are complicated by the softening magnon, which tends to merge with other low-energy features. This is

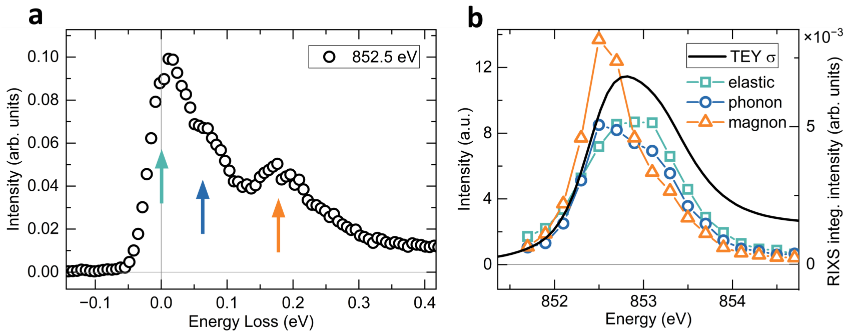

Figure S5 (a) RIXS spectrum at the magnon resonance, taken with π incident polarization. The arrows indicate the main spectral features in the low energy scale: the elastic line, the phonon peak and the magnon spectral weight. (b) Energy dependence of the different spectral features highlighted in panel (a) across the Ni L_3 edge. The integration intervals are [-0.03 eV, 0.03 eV], [0.03 eV, 0.1 eV] and [0.1 eV, 0.35 eV] for the elastic, phonon and magnon respectively.

testified by the large error bars on the damping at such values of Q. Nevertheless, our fitting results are in good agreement with literature [7, 8], including our very recent measurements on $NdNiO_2$ [9].

Bibliography

[1] C. Lichtensteiger, Interactivexrdfit: a new tool to simulate and fit x-ray diffractograms of oxide thin films and heterostructures. Journal of applied crystallography **51**(6), 1745–1751 (2018). https://doi.org/10.1107/S1600576718012840

[2] G. Krieger, A. Raji, L. Schlur, G. Versini, C. Bouillet, M. Lenertz, J. Robert, A. Gloter, N. Viart, D. Preziosi, Synthesis of infinite-layer nickelates and influence of the capping-layer on magnetotransport. Journal of Physics D: Applied Physics **56**, 024003 (2023). https://doi.org/10.1088/1361-6463/aca54a

[3] D. Preziosi, A. Sander, A. Barthélémy, M. Bibes, Reproducibility and off-stoichiometry issues in nickelate thin films grown by pulsed laser deposition. AIP Adv. **7**(1), 015210 (2017). https://doi.org/10.1063/1.4975307

[4] E. Breckenfeld, Z. Chen, A.R. Damodaran, L.W. Martin, Effects of nonequilibrium growth, nonstoichiometry, and film orientation on the metal-to-insulator transition in $NdNiO_3$ thin films. ACS Applied Materials and Interfaces **6**, 22436–22444 (2014). https://doi.org/10.1021/am506436s

[5] A. Raji, A. Gutiérrez-Llorente, D. Zhang, X. Li, M. Bibes, L. Iglesias, J.P. Rueff, A. Gloter, Unraveling P-Type and N-Type Interfaces in Superconducting Infinite-Layer Nickelate Thin Films. Advanced Functional Materials **34**, 2409930 (2024). https://doi.org/https://doi.org/10.1002/adfm.202409930

[6] K. Lee, B.H. Goodge, D. Li, M. Osada, B.Y. Wang, Y. Cui, L.F. Kourkoutis, H.Y. Hwang, Aspects of the synthesis of thin film superconducting infinite-layer nickelates. APL Mater. **8**(4), 41107 (2020). https://doi.org/10.1063/5.0005103

[7] H. Lu, M. Rossi, A. Nag, M. Osada, D.F. Li, K. Lee, B.Y. Wang, M. Garcia-Fernandez, S. Agrestini, Z.X. Shen, E.M. Been, B. Moritz, T.P. Devereaux, J. Zaanen, H.Y. Hwang, K.J. Zhou, W.S. Lee, Magnetic excitations in infinite-layer nickelates. Science **373**(6551), 213–216 (2021). https://doi.org/10.1126/science.abd7726

[8] M. Rossi, H. Lu, K. Lee, B. Goodge, J. Choi, M. Osada, Y. Lee, D. Li, B. Wang, D. Jost, et al., Universal orbital and magnetic structures in infinite-layer nickelates. Physical Review B **109**(2), 024512 (2024). https://doi.org/10.1103/PhysRevB.109.024512

[9] F. Rosa, L. Martinelli, G. Krieger, L. Braicovich, N.B. Brookes, G. Merzoni, M. Moretti Sala, F. Yakhou-Harris, R. Arpaia, D. Preziosi, et al., Spin excitations in $Nd_{1-x}Sr_xNiO_2$ and $YBa_2Cu_3O_{7-\delta}$: the influence of Hubbard U. Physical Review B **110**(22), 224431 (2024). https://doi.org/10.1103/PhysRevB.110.224431
